# Minimising Blood Stream Infection: Developing New Materials for Intravascular Catheters

**DOI:** 10.3390/medicines7090049

**Published:** 2020-08-26

**Authors:** Charnete Casimero, Todd Ruddock, Catherine Hegarty, Robert Barber, Amy Devine, James Davis

**Affiliations:** School of Engineering, Ulster University, Jordanstown BT37 0QB, Northern Ireland, UK; casimero-c@ulster.ac.uk (C.C.); ruddock-t1@ulster.ac.uk (T.R.); hegarty-c19@ulster.ac.uk (C.H.); barber-r@ulster.ac.uk (R.B.); devine-a14@ulster.ac.uk (A.D.)

**Keywords:** intravascular catheter, CRBSI, biofilm, CVC, antimicrobial, antifouling

## Abstract

Catheter related blood stream infection is an ever present hazard for those patients requiring venous access and particularly for those requiring long term medication. The implementation of more rigorous care bundles and greater adherence to aseptic techniques have yielded substantial reductions in infection rates but the latter is still far from acceptable and continues to place a heavy burden on patients and healthcare providers. While advances in engineering design and the arrival of functional materials hold considerable promise for the development of a new generation of catheters, many challenges remain. The aim of this review is to identify the issues that presently impact catheter performance and provide a critical evaluation of the design considerations that are emerging in the pursuit of these new catheter systems.

## 1. Introduction

Intravascular catheters are ubiquitous in contemporary care and it has been estimated that 30–80% of hospital patients will have a peripheral venous catheter (PVC) in place at some point during their stay [1,2,3]. While PVCs are by far the most commonplace, a wide range of catheter designs are employed to aid the delivery of life saving fluids and differ in terms of anticipated use. In contrast to the short-lived PVCs, central venous catheters (CVC) are designed for main vein access and mid to long-term (months/years) applications such as: the delivery of chemotherapy drugs, nutritional fluids and for haemodialysis. Used across every hospital unit as well as for outpatient management, CVCs are some of the most common indwelling medical devices of modern times with approximately 5 million catheters inserted annually in the US (cf. 330 million PVCs) [4,5]. While such devices provide ease of access to the vascular highways of the body, it must also be recognised that they can also serve as an entry point for life threatening infection [6]. It has been estimated that CVCs are some 64 times more likely to result in a catheter related blood stream infection (CRBSI) than other intravascular access devices [7].

Patients in intensive care units (ICUs) across the US are exposed to an estimated 15 million (catheter) days of CVC usage each year [8] from which up to 8% result in CRBSI [5,9]. CRBSI-induced sepsis accounts for 25% of annual haemodialysis patient mortality [10]. The US Centre for Disease Control and Prevention (CDC) estimates that a total of 250,000 blood stream infections are diagnosed each year [11] of which 80,000–120,000 are catheter-related [8,12]. While rates of attributable mortality vary from 12% to 25% for nosocomial CRBSIs [11,13,14], a meta-analysis concluded that an average of 3% of CRBSIs result in death [8]. Further complications can also arise such as intravascular thrombosis and endocarditis leading to myocardial infarction or stroke [15]. It is of little surprise that the consequences of CRBSI results in a considerable economic burden on both the healthcare provider and patient. The attributable cost per infection in the US varies considerably based on infection type and healthcare factors, costs are reported to range from USD 3700 to USD 56,000 per patient, with an upper estimate of USD 2.3 billion spent on CRBSIs arising from CVC use annually [8,16].

The combined morbidity, mortality and economic burden posed by CRBSIs, is an ever present concern among clinicians and has prompted much effort in the search for more effective frontline procedures (i.e., aseptic technique education and prophylactic antimicrobial lock systems) [17,18,19], but there has also been radical rethinks of the design and the development of new catheter components, materials and smart sensing solutions. There has been substantial progress in these areas in recent years but, despite concerted efforts in education and the implementation of care bundles, bacterial contamination and the resulting CRBSI remains problematic. As such, there are considerable opportunities for recent advances in materials and sensing technologies to complement improved clinical practice in providing a more integrated solution. The provision of materials that minimise the adherence of bacteria to the intraluminal space, provide antimicrobial action, and sensors that can proactively monitor the condition of the line herald a new generation of smart catheter systems that aim to eliminate infection or provide early warning diagnostics. The aim of this review is to provide a critical evaluation of the design considerations that are emerging in the pursuit of these new catheter systems. While the focus here is on CVCs, it should be recognised that bacterial contamination is commonplace and much of the discussion here should be transferrable to a range of devices.

## 2. Catheter Components

**Figure 1:**
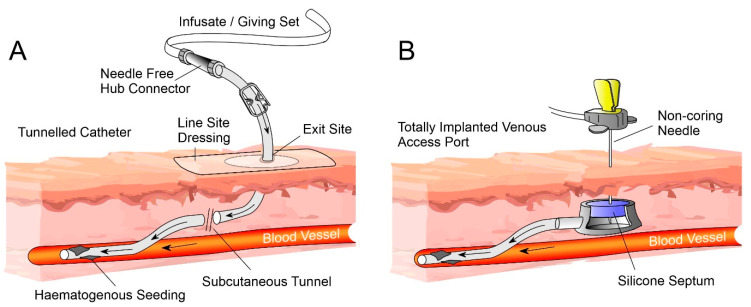
(**A**) Tunnelled central venous catheter and associated components. (**B**) Totally implantable venous access port.

There are a variety of CVC subsets available, the most common of which are: non-tunnelled, tunnelled, peripherally inserted central catheters (PICC) and totally implantable venous access ports (TIVAPs) [20]. The main components of a central venous catheter and the implanted venous access ports are highlighted in Figure 1A,B, respectively. In the case of CVCs, these are typically inserted through the jugular, subclavian or femoral veins to either the superior vena cava, right atrium or inferior vena cava of the heart [20,21].

Selection of the catheter system is invariably based on patient-specific factors such as: purpose, anticipated lifetime and the frequency with which the catheter will need to be accessed. It can be anticipated that the longer the catheter is in place, the greater the risk of infection. It is of little surprise therefore that tunnelled CVCs and TIVAPs which are intended for long term use (typically months to years) dominate the CRBSI literature. While TIVAPs are primarily designed for periodic/infrequent applications (i.e., haemodialysis) [22], tunnelled CVCs are generally targeted at those interventions where regular administration of fluids, medication, parenteral nutrition or the aspiration of blood is required. As such, the frequency with which the needle free connector (NFC) is manipulated can be particularly problematic with the majority of the infections arising as a consequence of its contamination [23,24].

**Figure 2:**
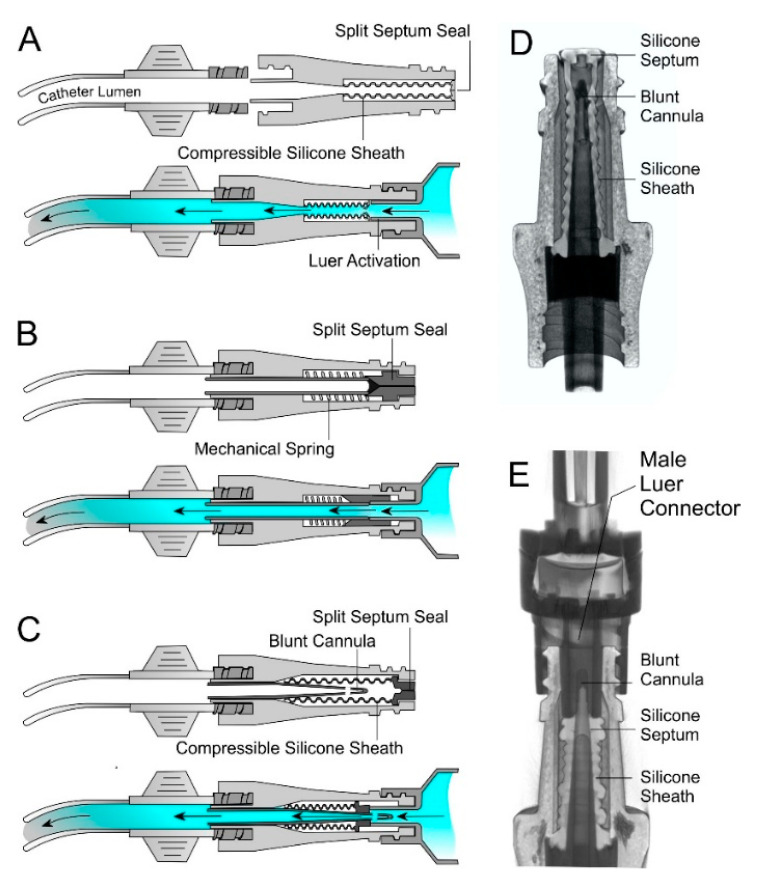
Needle free connectors based on (**A**) simple split septum, (**B**) mechanical spring compression and (**C**) blunt cannula. Computerised tomography scans of the internal components of an intensive care unit (ICU) Medical Clave™ connector before (**D**) and after connection to a giving set with a Luer connector (**E**).

A multitude of NFCs are available commercially but most share common design features relating to the access port. In almost all cases, access to the catheter is activated through the insertion of a male Luer connector (from a fluid giving set or syringe) which causes the deformation of a silicone septum and therein provides access to the catheter line [25,26,27]. Three of the more common approaches are highlighted in Figure 2. While they differ in terms of internal mechanism, most rely on a split septum design which, when disconnected, acts as a physical barrier to the entry of bacteria. Solid sealed silicone surfaces (BD Max Zero™) are also available and, while these potentially reduce the surface crevices through which bacteria can adhere, they still rely on the Luer activated displacement/compression of the silicone cap within the device to enable fluid flow to or from the line.

A variety of engineering features have been implemented in recent years as a means of improving the performance of such devices in terms of haemocompatibility and in reducing the potential for CRBSI. The presence of blood within the fluid pathway of an NFC can result in haemolysis of the red blood cells which increases the risk of a fibrin clot leading to occlusion and ultimately prevents fluid transfer. Moreover, it provides a pool of nutrients that can promote the growth of bacteria [25]. Body movements (muscle flexing, respiration, coughing etc.) and clamping of the catheter can all induce changes in the mechanical and physiological pressure within the catheter that can serve to push blood along the line [28,29]. It has been estimated that even the smallest blood reflux (4–30 mL) can result in fibrin activation and occlude the inner pathways of the NFC [25]. Removing blood from the NFC is a critical concern and there has been an increasing shift from opaque NFC structures to more transparent polymers that can enable visual inspection of the internal working of the hub. The movement of blood within the catheter and its propensity to travel (reflux) to the needle free hub upon the insertion and disconnection of the external Luer is however dependent on the design of the NFC hub [25,26].

It is of little surprise therefore that understanding the operation of these systems can be a major factor in minimising the risk of infection. Depending on the displacement of blood upon insertion/disconnection, hubs are generally classified as: negative, neutral, positive and anti-reflux. A summary of the various designs and their mechanism is provided in Table 1.

It can be seen from Table 1 that in the case of negative and positive displacement systems, there is a recommended clamping procedure associated with the use of these systems to prevent the inadvertent reflux of blood. There is, however, an assumption that the clinical staff (or the patient in the case of known as home parenteral nutrition (HPN)) are aware of the mode through which a particular NFC operates. Hadaway (2011), in a survey of healthcare workers, found that of 554 responses, some 25% were unaware of whether the NFC used in the CVC line they were managing was positive, negative or neutral [30]. Moreover, 47% were unsure as to the correct approach to the flushing and clamping procedures associated with a particular NFC with the situation being compounded by the presence of multiple NFC brand variants in use within a given institution [27,30,31]. The early introduction of NFCs was characterised by an increase in CRBSI and a lack of appropriate training in device operation has often been cited as a contributing factor. This has been corroborated in instances where an institution has switched NFC brands and recorded an increase in CRBSI rates only to find the latter returned to previously lower levels when resuming use of their original NFC system [31]. While improvements in the physical design of NFCs will undoubtedly aid approaches to the prevention of CRBSI, the continuing prevalence of the latter however highlights that there is much still to be done.

## 3. Pathogens, Colonisation, Biofilms and Infection

In general, catheter related infection occurs mainly through two mechanisms—migration of adventitious skin pathogens (i.e., *S. aureus*) along the external surfaces of the polymer tubing through the cutaneous tract to the blood stream (extraluminal) and, as noted in the previous section, ingress via the needle free connector hub (intraluminal) [24]. In either case, upon contact with the blood, the microbes interact with fibrin to yield an adherent biofilm which promotes microbial colonisation and furthers the spread of the organisms. Extraluminal contamination has historically been more common in short term intravascular devices (peripheral venous/arterial catheters and non-cuffed/non-tunnelled CVCs) and typically arises through issues encountered during insertion/implantation [24].

Breaching the skin barrier to enable the insertion of a medical device inevitably provides an opportunity for skin flora or adventitious contaminants from the healthcare environment to gain access to the underlying tissues and from there sets a foundation for subsequent infection. Such issues are not restricted to intravascular access devices but have become increasingly common with cardiovascular implantable electronic devices (CEID) such as pacemakers, cardioverter–defibrillators, cardiac resynchronisation devices etc. This is compounded by the increasing longevity of the patient cohorts where the number of surgical interventions (revisions, extractions and upgrades) have increased substantially year on year [32,33]. As the prime risk occurs at the time of insertion, it is of little surprise that repeated surgical replacement of a CVC (or the CEID) increases the risk of infection. The implementation of catheter care initiatives during the insertion of CVCs (such as the Keystone Central Line Bundle [34] and Epic3 guidelines [19]) have however led to significant improvements in outcomes and, in the US, has led to reductions in insertion related infections [23,25,35].

**Figure 3:**
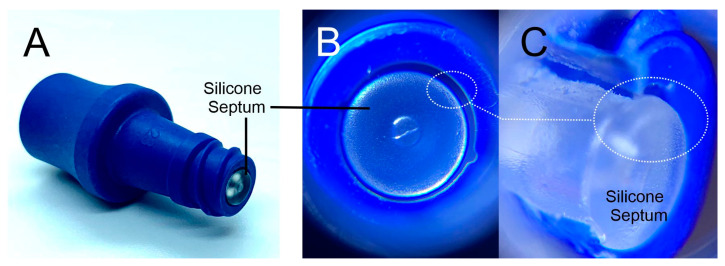
ICU Medical Clave™ needle free connector (**A**). Optical image of the septum (**B**) and cut through section (**C**) highlighting gaps in the structure.

In contrast, contamination of the NFC and internal lumen tends to occur post-operatively as a consequence of failures in the aseptic manipulation of the connecting hub prior to the administration of fluids or aspiration of blood. Intraluminal colonisation tends to be predominant in those devices intended for longer term function such as cuffed Hickman and Broviac type catheters, cuffed haemodialysis CVCs, TIVAPs, and PICC systems [36]. It is of little surprise that the increased duration of placement and frequency with which such lines are accessed will also increase the risk of CRBSI where there will be more opportunities for the intraluminal migration of planktonic (free-swimming) bacteria arising from a contaminated hub to the bloodstream [7,24]. Examination of the microbial contamination of NFCs after periods of non-use found that colony forming units (CFU) varied from 15 to 1000 CFU which, if improperly disinfected, would be sufficient to induce colonisation of the catheter and result in bacteraemia [31,37,38]. Potential trouble spots in the use of the NFC, in terms of disinfection, relate to the point where the sterile Luer connector or syringe contacts the surfaces of the NFC—mainly the septum, side threads and side surfaces (between septum and NFC structure) [23]. The presence of grooves/gaps either in the core design between septum seal and housing (as highlighted in Figure 3 for the Clave™ NFC), or as a result of repeated use (i.e., abrasion or other physical damage) can all influence the ease with which disinfection can be achieved [26,27,30,39].

While the majority of CRBSI are known to originate from issues in aseptic manipulation, two other sources of infection also need to be considered which are effectively independent of any attempt at external decontamination of the catheter or the NFC. Contamination of the fluid to be infused will effectively bypass any aseptic precautions employed during administration and offers microbes unimpeded access to the bloodstream [40]. Fortunately, such occurrences are exceedingly rare. Haematogenous seeding occurs when pathogens already present in the bloodstream as a consequence of local infection (i.e., pneumonia) encounter the foreign extraluminal surface and then subsequently colonise it (indicated in Figure 1A). Though it must also be recognised that a contaminated catheter can also serve as a potential seeding source for the contamination of other intravascular devices [41]. The pathogens most commonly cultured from infected CVCs are listed in Table 2. *Staphylococcus aureus* and coagulase-negative staphylococci (typically *S. epidermidis*) are the two pathogens most frequently isolated in CRBSI cases, with *S. aureus* responsible for between 10% and 25% of infections [42,43].

The introduction of bacteria to the lumen of the catheter line will inevitably result in the formation of a biofilm (largely polysaccharide in nature) which serves as a foundation for the sustained growth of the microbes and ultimately as a latent infective source [44,45]. The film itself is an extracellular 3D network that protects the emerging communities through serving as a physical barrier against the body’s intrinsic immune response (phagocytes) and limits the diffusion of antibiotics. These protective qualities can be particularly problematic when attempting to salvage a catheter (rather than its direct replacement) where the presence of any surviving bacteria within the biofilm can lead to a resumption of the infection [45]. As such, conventional antimicrobial therapies require concentrations some 100–1000 times greater than the normal minimum inhibitory concentrations (MIC) to be applied and the dosage maintained over longer durations in order to eradicate the biofilm [46]. A more worrisome issue is that in aiding bacterial reproduction, the biofilm can aid the alteration of bacterial gene expression, leading to mutations and modifications in the physiology of the pathogenic antigens, limiting immune and drug response and contributing to antimicrobial resistant species [47,48].

Extra and endoluminal colonisation rates are dependent on the interaction between physiological properties of the pathogen and the surface characteristics (i.e., hydrophobicity/hydrophilicity) of the catheter [49]. The initial adhesion of pathogens is improved by the initial formation of a conditioning film (comprised of platelets and plasma proteins such as albumin, fibrinogen, and fibronectin) that binds to the luminal surfaces [50]. Pathogens will more readily adhere to this film than to the bare catheter material itself [49] and, once attached, to the luminal surface, will rapidly establish a biofilm. It is of little surprise that there have been extensive efforts to modify the surface of the polymers used in the production of both the catheters and NFCs such that the initial deposition of the conditioning film is impeded.

## 4. Current Practice

### 4.1. Disinfection

Alcohol/antimicrobial wipes are widely employed as the primary anti-infective measure in the management of catheter lines and decontamination of NFCs. Alcohol wipes (typically 70% isopropyl alcohol (IPA)) are the most commonly applied measure and their biocide activity relies on their ability to dehydrate the bacterial cell—both during the application and as the alcohol evaporates [51,52]. While the use of the alcohol alone can be effective, its veracity can be greatly enhanced by the presence of an appropriate antimicrobial agent (chlorhexidine or povidone iodine) where the disinfection mixture exploits the immediacy of the alcohol and sustained action of the antimicrobial agent [23,30,53,54,55,56].

Alcohol disinfection is not however fool proof and will always be subject to human factors (time allocated to the procedure, friction applied etc.) and device designs (ability to penetrate the device crevices) and there remains a contentious debate as to whether complete removal of bacteria from NFCs is in fact possible [52,57,58,59,60]. Menyhay and Maki (2008) in an in vitro study of 30 NFCs contaminated with *E. faecalis* and then subsequently disinfected with 70% IPA, found that 67% of the NFCs continued to transmit microbial contaminants (440–25,000 CFU) [52]. The time allocated to disinfection appears to be a significant factor with studies by Kaler and Chin [52] finding that 15 and 60 s cleaning cycles with 70% IPA eliminated all organisms whereas studies by Smith et al. (2012), Simmons et al. (2011) and Rupp et al. (2012) highlighted that short to moderate cleansing (3–15 s), while decreasing bacterial load, were less effective at total decontamination [57,58,59]. There is nevertheless considerable variability and contrasting results, as befits the nature of human intervention involving “scrubbing the hub”. The UK EPIC3 report on the evidence-based evaluations of an expert panel have recommended that NFCs be disinfected with 70% alcoholic chlorhexidine with the application of friction pre and post access [19].

### 4.2. Education and Aseptic Techniques

Training at the core of the management of CVCs—from implantation to the day to day care of the line but the reliance on human compliance and adherence to the main tenets of aseptic manipulation can be an inherently variable phenomenon [61,62,63,64]. While the introduction of care bundles has led to significant gains, it must also be noted that improved compliance rates are seldom universal. A recent study by Jeong et al. (2013) revealed compliance rates were only 37% after the intervention [63] and a recent meta-analysis by Ista et al. (2016) highlighted that total compliance is essentially unattainable [18].

While the significance of disinfection of the NFC prior to accessing the catheter has long been recognised, it is surprising that it remains a common point of failure [23,30,64]. Patients with long term CVCs undergoing total parenteral nutrition (TPN) are trained to administer their nutrition at home, or have it administered at home on their behalf, known as home parenteral nutrition (HPN). In a study conducted by Bond et al. (2018), 16% of HPN patients contracted at least one CRBSI, accounting for 0.31 CRBSI per 1000 catheter days [65]. Out of these 16%, the rate of CRBSI per 1000 catheter days was 0.27 when HPN was administered by a trained home care nurse, compared to 0.342 and 0.320 when self-administered or administered by a non-medical carer (such as a family member), respectively. It is noteworthy that although there are gains in having a trained caregiver—the benefits are only marginally better than having the patient manage the line. There is little doubt that more attention is required for training in aseptic access and maintenance [66].

### 4.3. Catheter Locks

A second line of defence in preventing bacterial ingress rests on the use of catheter locks. The latter is typically used where the catheter is not being used and the line is flushed with saline. This has the primary purpose of removing blood from the line such that occlusion and bacterial growth are minimised (discussed in Section 2) [26,27,30,67]. While the use of a saline flush is standard, other components can also be introduced and perform a variety of roles: anti-occlusion (heparin), antibiotic (vancomycin, gentamicin) and antimicrobial (citrate, ethanol and taurolidine) [68,69,70]. Vancomycin and gentamicin are generally reserved for therapeutic measures once a CRBSI has been diagnosed [22,46,71,72,73]. In contrast, heparin, citrate and, increasingly, taurolidine are used prophylactically [9,74,75,76]. There is no standard recommendation as to the use of catheter locks and their over-arching function is to maintain the integrity of the line. An extensive literature based on their application and investigations of their efficacy has emerged in recent years but a detailed discussion of their individual use is beyond the scope of the present study and the reader is directed to more comprehensive reviews [68,69,70].

### 4.4. Barrier Caps

**Figure 4:**
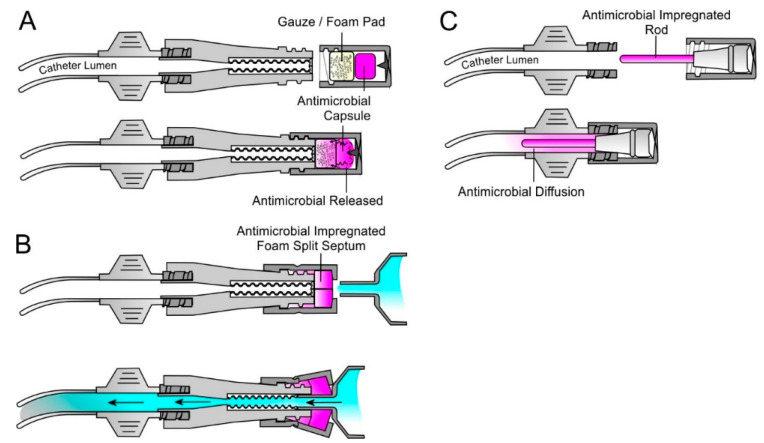
Needle free connector barrier caps based on simple foam insert (**A**), an encapsulating split septum (**B**) and the Clearguard^®^ terminal cap (**C**).

In most cases, the silicone septum is the main physical barrier preventing the entry of microorganisms to the catheter lumen. Disinfection, while widely recognised as critical, still falls foul of issues relating to adherence and on the vagaries of the person performing the cleansing process [23,25,27]. Passive NFC caps have come more to the fore in recent years through providing a passive means of continuous disinfection whilst the catheter is not in use [77,78,79,80,81,82,83,84]. A number of different approaches have been taken in the design of the disinfectant barrier cap and their mode of operation is summarised in Figure 4. The simplest approach is the incorporation of a foam/gauze insert soaked with 70% IPA (i.e., 3M Curos™, SwabCap^®^, Site-Scrub^®^) or one that contains an antimicrobial (povidone iodine or alcoholic chlorhexidine) which is threaded onto the Luer connector when the line is not in use. As the foam pad contacts the silicone septum, the twisting of the cap provides a modicum of friction which can aid in the removal of the bacteria. Menyhay et al. (2008) have reported on a refinement of the basic design whereby a two-part system is employed with the antimicrobial contained within a capsule (Figure 4A) [52]. As the cap is threaded onto the Luer, the capsule is forced into contact with a spike at the top of the cap which pierces the capsule releasing the antimicrobial into the foam. The core advantage of this and similar systems are that their application is relatively independent of the NFC manufacturer and simply require a Luer connector. An alternative design has been proposed by Buchmann et al. (2009) whereby the cap encapsulates the entire terminal end of the NFC including the thread (Figure 4B) and, in contrast to the simple insert cap highlighted in Figure 4A, is intended to remain attached during flush procedures [85]. Mariyaselvam et al. (2015) reported that contamination of syringe tips is an often overlooked factor in the development of CRBSI and hence retention of the antimicrobial foam as a secondary septum could aid in the decontamination of the tips prior to entering the NFC [86].

In both designs, the barrier cap does not interact directly with the catheter lumen but aims to disinfect the silicone septum and surrounding area and are intended for applications where the frequency of access may be high. ClearGuard^®^, in contrast to the previous designs, relies upon a chlorhexidine impregnated rod that is inserted directly into the end of catheter lumen in place of the NFC connector (Figure 4C). The chlorhexidine diffuses into the intraluminal space and has been shown to be highly effective at reducing CRBSI risk within haemodialysis patients—a cohort that has hitherto been characterised with high rates of infection [82].

## 5. Catheter Designs and Antimicrobial Mechanisms

**Figure 5:**
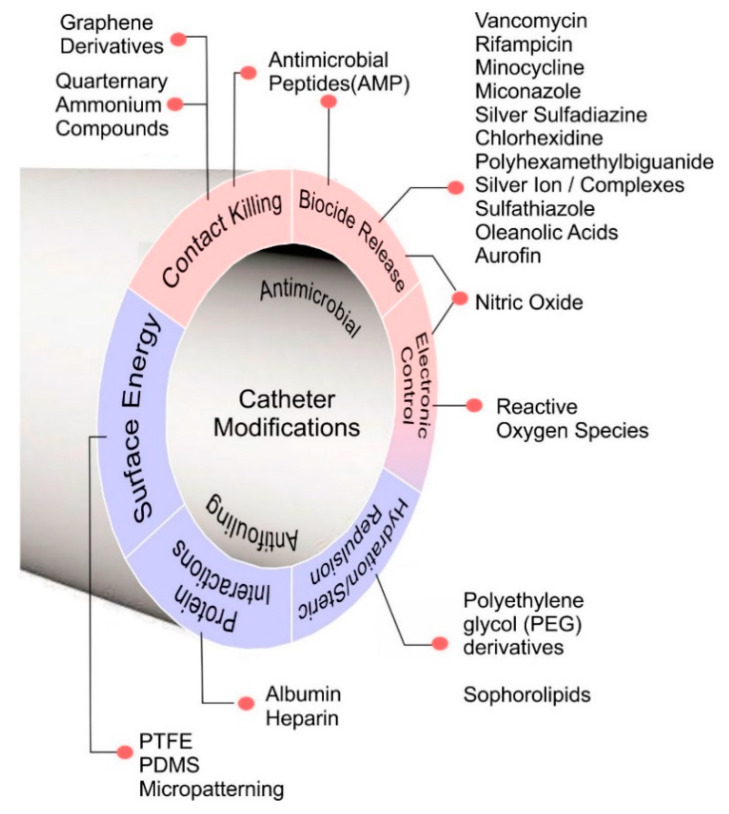
Material approaches to counter bacterial colonisation of central venous catheters.

The provision of training programmes to educate clinicians and patients in the maintenance of vascular access lines is a critical frontline response that can dramatically lower the risk of CRBSI but, as noted earlier in Section 4.2, achieving 100% compliance and prevention is unlikely. The provision of antibacterial caps is another advance which has demonstrated considerable gains but, as with conventional aseptic practice, there remains a human element in their successful application as well as several contentious cost issues that can be a barrier to their widespread adoption. The development of materials resistant to antimicrobial colonisation has long been regarded as a means through which to counter the potential lapses in practice and where elimination of the propensity to form a biofilm is the principal goal. The majority of the research efforts targeted at this problem are generally directed towards materials that possess antimicrobial and/or antifouling properties and a summary of the various strategies are highlighted in Figure 5.

Tunnelled CVCs, non-tunnelled CVCs and PICC-lines are typically made of polyurethane (PU) or silicone materials, with tunnelled CVC cuffs composed of polyethylene terephthalate (PET) [87]. The preference for PU stems from the versatility with which the physical and chemical properties of the material can be manipulated through judicious choice of the monomers [88,89,90]. In general, PU is prepared from the reaction of hydroxyl and isocyanate groups to yield the carbamate linkage (the urethane) and, with a large array of commercially available polyols and polyisocyanates bearing a range of chemical functionalities from which to choose [89,90], it is of little surprise that the properties of the polymer can be tuned to particular applications. The polymer properties can be critical in influencing the processability of the material and its resulting mechanical performance and biocompatibility [88,91,92,93]. Critically, the ability to alter the chemical functionality allows for modifications to the hydrophilicity/hydrophobicity of the surface and, through the presence of reactive chemical side chains, provides the ability to further tailor the catheter interface with antimicrobial/antifouling features [88,93,94,95,96,97,98,99,100,101].

### 5.1. Biocide Release

The antimicrobial systems employed on both the extra and endoluminal surfaces aim to kill the bacteria (or at the very least inhibit further growth) and a number of different mechanisms have been evaluated. Drug eluting materials in which the biocidal agent is released passively into the lumen represent the most common approach (as listed in Figure 5) and cover a wide variety of chemical species [95,102,103,104,105,106,107,108,109,110]. Incorporation within the catheter can be achieved through simple adsorption of the active agent but, more recent strategies have involved electrostatic interactions with surfactants and polyelectrolyte systems to yield more coherent and stable coatings. Thermally stable biocides such as silver ions/complexes can be melt processed along with the polymer used in the production of the catheter such that the antimicrobial is homogeneously distributed throughout the material. Alternatively, exposing the catheter surfaces to a suitable solvent can induce swelling of the polymer and impregnation/incorporation of the biocide at low temperature. Some of the commercial antimicrobial catheter systems and their characteristics are compared in Table 3.

The efficacy of employing antimicrobial catheters has been widely studied for most of the systems outlined in Table 3 or their equivalent, and there is a substantial body of the literature which has found marked improvements over the use of unmodified catheters. There have also been numerous reports that have found little benefit. It must be noted that there are a large number of factors involved in the maintenance of CVCs (as noted in earlier sections) which can make comparisons between the different systems and between coated/uncoated challenging. Nevertheless, a number of systematic reviews have conducted meta analyses of the available literature and there is substantive evidence that the implementation of catheters coated/impregnated with chlorhexidine/silver sulfadiazine and antibiotics (5-fluorouracil, vancomycin, benzalkonium chloride, teicoplanin, miconazole/rifampicin, minocycline, and minocycline/rifampicin) were associated with lower incidences of catheter colonization and had the greatest potential to reduce the incidence of CRBSIs per 1000 catheter days. In contrast, the efficacy of silver impregnated systems is much more contentious and, in many cases, fail to yield statistically significant results.

The Healthcare Infection Control Practices Advisory Committee (HICPAC) recommend the use of a CVC impregnated with chlorhexidine-silver sulfadiazine (CSS) or minocycline-rifampicin (MR) in patients with at least five consecutive days of catheterisation [111]. A recent systematic survey by Lai et al. (2016) found that the most effective system within this class of material was the rifampicin but it must be noted that such studies were of limited duration and their efficacy in CVCs destined for long term placement is questionable [112]. As such, most of the commercial systems highlighted in Table 3 recommend a maximum dwell time. A core issue is the limited repository of the drug within the polymer or coating such that release does not simply terminate after a given time period but results in sub lethal doses being administered which can facilitate the development of antimicrobial resistance.

Putting the issue of dwell time aside, the release of antibiotic moieties (i.e., rifampicin) have a long history but the increasing threat of bacterial resistance has driven considerable effort to examine alternative antimicrobial agents. Various antimicrobial peptides [100,108,113,114,115], guanidine derivatives (i.e., poly hexamethyl biguanide, polyarginines) [103,104], quaternary ammonium compounds [96,98], nitric oxide precursors [105,106,116,117], silver [118,119,120] and a host of other small molecules/metal ions or nanoparticles [107,110,118,121,122,123,124] with possible biocidal activity have all been investigated as potential modifiers for use in catheters and, while these invariably impact bacterial colonisation, they have yet to make the leap to commercial exploitation and/or substantive clinical trials.

### 5.2. Contact Kill Systems

Contact killing of bacteria, in contrast to passive elution, relies on the immobilisation of the antimicrobial at the catheter surface and, as such, sets out to present a lethal barrier to the microbes attempting to colonise the catheter surfaces [96,98,100,104,114,115,119,125,126]. Grafting through plasma processes, polymerisation of a biocide functionalised monomer, covalent linkage (i.e., click chemistry) onto side chains or the deposition of insoluble layers are common techniques through which the catheter interface can be functionalised. Quaternary ammonium compounds (QACs), guanidine derivatives, antimicrobial peptides (AMPs) and, more recently, graphene/graphene oxide [125] systems have all been evaluated as contact killing agents. In terms of QAC and AMPs, their cationic functionality and ability to disrupt the phospholipid bilayer are the main weapons through which they attack the integrity of microbial cell wall/membrane. The mechanisms through which graphene and its various analogues work are more contentious though there is evidence to suggest that the edge planes of graphene platelets directly exert a membrane disruption effect. The in situ generation of reactive oxygen species (ROS) through redox cycling of quinoid functionalities in graphene oxide has also been shown to be a potential cytotoxic pathway [125].

As the active agents are immobilised at the surface of the catheter, the biocidal activity is, at least in a model system, capable of being maintained indefinitely. Unfortunately, the need for direct contact between the agent and the bacteria can also be a significant limitation. The deposition of conditioning films or macromolecular debris (i.e., from dead bacteria or non-specific binding of proteins) along the lumen can negate the antimicrobial effects through preventing these killing interactions. Although the contact killing approach, like the drug elution systems, appears to offer only short-term activity, it should be noted that, unlike the latter, the underpinning mechanism has no impact on emerging antimicrobial resistance. Applied in isolation, the contact approach is clearly limited by fouling but, if the latter were removed, then it could be envisaged that long term effectiveness could be achieved.

### 5.3. Surface Hydration/Hydrophilicity

Prevention of fouling has been the second main route through which to avoid biofilm formation and minimise the risk of both CRBSI [97,99,114,127] and catheter related thrombotic complications [128,129,130,131]. The latter can be categorised into four types: mural thrombosis, ball-valve-thrombosis, intraluminal thrombotic occlusion and, the most common cause, pericatheter sheath [128,129,130,131]. In short, the presence of the fibrin sleeve is due to catheter insertion causing local venous injury, leading to the deposition of fibrin on the catheter surface and subsequent intraluminal growth of endothelial and smooth muscles within hours after CVC insertion [130]. This in turn could lead to blood flow reduction which further increases the risk of endoluminal cellular attachment and thus thrombus formation [129,130]. Further movement of the catheter within the vein causes endothelial erosions which prompt the formation of mural thrombosis within the catheter lumen [128,130]. On the other hand, a thrombus on the catheter tip could lead to ball-valve thrombosis where infusion can still occur, but fluid aspiration is impeded [128,130].

As indicated in Figure 5, a number of strategies aim to prevent fouling. Historically, the modulation of hydration and steric interactions were among the first approaches and typically exploit polyethylene glycol (PEG) derivatives tethered at the polymer–solution interface [99]. The rationale here is to control the hydrophilicity of the polymer interface to create a tightly bound water layer. This alters the thermodynamics of adhesion through making it both physically and energetically less favourable for the adsorption of proteins. While PEG derivatives have dominated the early literature, alternative systems incorporating zwitterionic moieties (i.e., polysulfobetaine) have been used in PICC lines and shown to reduce the adhesion of bacteria and the onset of thrombosis [132]. Instead of hydrogen bonding, zwitterionic based coatings use electrostatic interactions to create the hydration layer [131,133]. Roth et al. (2020) demonstrated the use of branched polyethyleneimine (PEI) modified polyurethane as a means of reducing the coefficient of friction and haemolysis ratio providing a material with considerable antithrombogenic properties [131]. The intrinsic inertness of silicone-based catheter materials can be problematic, but plasma treatment can enable the introduction of more reactive surface functionalities onto which antifouling coatings can be anchored. This was adopted by Blanco et al. (2014) who demonstrated the use of a laccase/phenolic/sulfobetaine mixture to yield a zwitterionic film tethered to a plasma aminated silicone substrate [133]. The system, although initially targeting urinary catheters, demonstrated considerable antifouling capabilities which could however be translated to intravascular systems. The novelty of the biocatalytic film formation is clear but it could be argued that the complexity of the approach would be a detractor from more widespread adoption.

It is clear that the adaptation and incorporation of the antifouling film systems could have a significant impact on mortality as it has been estimated that some 20% to 40% catheters develop pericatheter thrombus or fibrin sheath [134]. The latter predisposes the patient to infection and increases the risk of thrombosis [135,136] and, if detachment occurs, the possibility of potentially fatal thromboembolism [137].

### 5.4. Protein Layer Interactions

The use of a protein coat to prevent the adhesion of other proteins can appear counter intuitive but such interactions are typified by the precoating of catheter surfaces with albumin (a relatively benign protein). This approach has been shown to markedly reduce the deposition of proteins that would otherwise adhere and contribute to biofilm formation [138,139]. The effectiveness of such an approach is however relatively short term as prolonged contact with blood eventually leads to the removal of the albumin. The use of heparin as a catheter lock is well established where it is employed to prevent thrombus and occlusion of the line [67,68,69,70,84]; catheter surfaces coated with the molecule have also exhibited marked resistance to non-specific protein fouling [92,119,140]. Several conflicting mechanisms for this action have been suggested (electrostatic repulsion, protein specific interactions, inhibition of bacterial adhesions etc.) but much remains to be done in order to elucidate whether they act in concert or if one predominates. The use of heparin coatings, as with its inclusion in lock solutions, can also give rise to concerns over sensitivity [141,142,143].

### 5.5. Surface Energy

The ideal solution would be to have the catheter composed of a material that minimises adhesion without the complexities of extensive surface modification. The adoption of materials possessing low surface energy has been proffered as one route through which to tackle biofilm formation and is typified by hydrophobic fluoropolymers (PTFE) and silicones (PDMS) [139,144,145,146]. Despite possessing very low surface energy (<25 mN/m), their effectiveness at preventing the non-specific adhesion of proteins is contentious with a number of investigations offering conflicting evidence. Where such polymers have found success, it has been suggested that passivation by albumin is the main factor in hindering cell attachment rather than the intrinsic low energy properties of the polymer substrate [139,144]. The exploitation of hydrophobicity in anti-adhesion contexts may however require a more nuanced application and there have been some notable advances through manipulating the surface topography [97,147]. Increasing surface roughness or the introduction of specific patterning inspired by biological materials (i.e., Sharklet), have shown to provide superhydrophobic coatings that are effective against *E. coli* and *S. aureus* [97]. The translation of the technology to catheter systems to prevent extra and intra luminal colonisation may be more challenging and, at present, such approaches remain speculative.

### 5.6. Smart/Electronic Materials

The majority of materials research aimed at combatting CRBSIs tend to focus on the modification of catheter surfaces and, while many of the strategies have shown to be effective in the short term, almost all succumb to fouling with increased dwell time and a loss of activity. Advances in electronics have seen some interest in the development of “smart” materials that can detect the presence of a biofilm or, upon activation by an appropriate stimulus, release a biocidal agent into the lumen. Such research is still in its infancy but could herald a wholly new avenue for tackling CRBSI.

Li et al. (2014) were among the first to consider the introduction of sensors within the catheter line as a means of detecting the presence of bacteria [148]. Their approach relied upon the use of carbon fibre filaments (possessing the dimensional properties necessary for insertion with a typical catheter lumen) serving as electrodes which could measure changes in the pH of the intraluminal space as a consequence of bacterial growth. This was based on examining the change in the voltammetric response of uric acid (present within the blood) with changes in pH. An alternative approach by Davis et al. (2013) took the system further through the use of polymer modified electrodes in which pH dependent redox polymers based on plumbagin were used to indirectly measure pH and removed the dependence on the endogenous urate [149]. This was followed by Casimero et al. (2018) with a poly(flavin) system, again exploiting the redox transition of the immobilised flavin to gauge pH changes caused by bacterial growth [150]. These reagentless sensors, while capable of measuring pH within complex bacterial environments, also possessed the advantageous attribute of being capable of catalysing the electroreduction of oxygen resulting in the generation of biocidal reactive oxygen species (ROS) [149]. The core mechanism of the polymer modified electrodes relies upon the pH dependence of quinoid redox interconversions and, in this respect, they could be considered analogous to some of the more recent investigations employing graphene oxide where similar transitions are associated with ROS. The main difference here being that the amount of ROS generated via the poly(plumbagin) could be controlled through manipulation of the electrode potential. Such work was however purely proof of concept and there is no supplementary data on their effectiveness in reducing/preventing bacterial colonisation [149].

Paredes et al. (2014) proposed a novel technique for in-line diagnosis of bloodstream infections through impedimetric biosensing [151]. The system incorporates an interdigitated microelectrode biosensor (IDM), wireless electronics and antenna to detect infection and then trigger an external alarm. Colonisation and subsequent biofilm growth on the IDM alters the capacitance, which is compared to a threshold generated using laboratory-based impedance spectroscopy, providing a preventative warning at the earliest signs of colonisation. The use of a label-free IDM provides the system with a lifetime of over 11 months, requiring only a 50 mAh coin-cell battery. A critical issue here, however, is an assumption that material on the surface of the electrode is a biofilm, when it could be the adsorption of macromolecular components intrinsic to blood. There is little doubt that the introduction of diagnostic systems that can inform the clinician (or patients self-administering their line) of the presence of contaminated NFCs could aid in the management of the CVC and optimise the use of hygiene care bundles.

**Figure 6:**
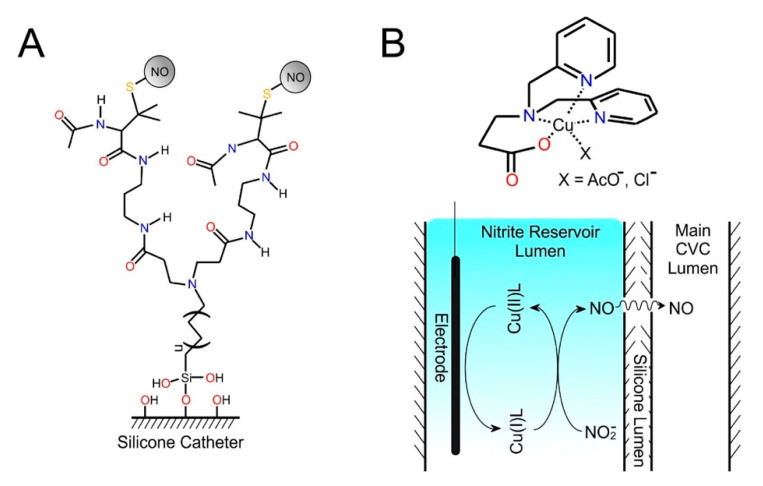
(**A**) Hyperbranched methacrylate-penicillamine based nitric oxide (NO) donors. (**B**) Dual lumen electrocatalytic release of nitric oxide using copper complexes.

The smart approach, as indicated previously, is not necessarily limited to diagnostics but can also be harnessed to yield an “on demand” antimicrobial action. In addition to the generation of ROS, reactive nitrogen species (RNS) have also been considered and several reports have targeted the selective release of nitric oxide (NO) as the principal weapon through which to prevent bacterial colonisation [105,106,116,117]. Such work builds on the fact that NO is a chemical transmitter which has a multitude of biochemical roles but, in this context, its status as a key player in minimising platelet adhesion whilst also acting as a broad spectrum antimicrobial is of greatest value. Numerous studies have investigated the use of NO donor molecules (i.e., diazoniumdiolates and S-nitrosothiols) immobilised at catheter surfaces [105,106]. In such cases, the mode of action following the passive release mechanism (through trans-nitrosation of other molecules within the matrix and/or homolytic cleavage) is common to conventional antimicrobial elution systems. Goudie et al. (2019) recently demonstrated the covalent linkage of N-acetyl penicillamine onto silicone catheters in which the thiol could be further functionalised to serve as a NO donor [106]. The use of branch (and hyper branched) methacrylate linkers as indicated in Figure 6A enables a greater density of NO to be stored at the interface with the polymeric network found to retain its anti-fouling properties even after the NO biocides have been delivered.

Mihu et al. (2017) have sought to take a different approach through the thermal reaction between nitrite and glucose during a sol–gel process [152]. This results in the in situ generation of NO and its subsequent entrapment with the sol–gel nanoparticle matrix. In contrast to the previous investigations involving donor molecules, NO is released directly from the nanoparticles. This has been shown to afford activity against bacteria with MRSA cellular growth decreased by 40% following incubation with 2.5 mg/mL NO-np, and by 50% at concentrations greater than 5 mg/mL. The viability of staphylococcal biofilms also reduced by 51.8% under the latter conditions. Furthermore, MRSA growth was seen to be reduced by 50% after 8 h of normal exposure (no incubation) with 2.5 mg/mL NO-np, remaining at these reduced levels after 24 h. The latter results indicate a similar level of success to antibiotic locks, with comparable dwell times. Impregnation of nitric oxide-releasing nanoparticles could prove to be a potential alternative to prophylactic locking, removing reliance on antibiotics and providing a broader effective range; but further research into their in vivo effects are required.

The electrochemical generation of NO represents a new stage in the evolution of active catheters and various reports have examined its electrocatalytic generation from nitrite [116,117]. A variety of copper complexes have been investigated as potential redox cyclers (indicated in Figure 6B). Both the copper complex and nitrite ion must be supplied to the system in order to facilitate the electrogeneration of NO as neither will be present in the IV fluids being delivered. This stands in marked contrast to the plumbagin (or graphene oxide) system where the catalyst can be immobilised at the electrode and the principal feedstock is oxygen which is already endogenous to both the blood and IV fluids being administered. In order to counter this, the authors have suggested the use of a dual lumen CVC in which one lumen is dedicated to the NO generation with the other for the IV fluid transfer. This separates the potentially harmful catalyst and nitrite from direct contact with the vascular system and relies upon the diffusion of electrogenerated NO across the silicone membrane separating the two fluid lines as indicated in Figure 6B. This is a much more complex arrangement from both procedural and instrumentational perspectives. However, the availability of a large reservoir of the nitrite feedstock could counter the issues of depletion and fouling that is common to most of the conventional antimicrobial/anti-adhesion approaches and prove to be effective on long CVC dwell times.

## 6. Conclusions

Catheter-related bloodstream infections have proven to be one of the most common nosocomial infections in the modern healthcare setting. For a condition that seems almost extraneous, they have a considerable impact on patient quality of life, up to the point of mortality, and pose a serious and undue economic burden on patients and health services. While there have been advances in each of the discussed areas, from improved aseptic techniques, new antibiotics and alternative lock therapies, these revolutions do not negate the core issues: aseptic techniques are only as reliable as the compliance of the user, systemic antibiotics are successful if you catch the symptoms early and the efficacy of antibiotic lock therapy is still up for debate.

Current practices for CRBSI diagnosis, prevention and management rely too much on therapeutic techniques, effectively waiting for patient quality of life to deteriorate before any action is taken. While there have been advances in catheter-sparing and prophylactic techniques, with the new guidelines promoting the use of impregnated catheters, and the improved variety, efficacy and range (outside of antibiotics) of antimicrobial locks looking promising, the impact on patient quality of life, cost and mortality is still too significant to declare these practices as viable solutions. There still remains a pressing need to develop a long-term solution for the proactive monitoring of catheters, a solution that will detect infection earlier or prevent it altogether.

## Figures and Tables

**Table 1:** Classification of needle free connector hubs in terms of fluid displacement.

Action	Negative	Neutral	Positive	Anti-Reflux
Disconnection	Blood refluxes into catheter	Blood refluxes into catheter	Fluid moves towards patient	Fluid restricted by diaphragm
Connection	Fluid moves toward patient	Fluid moves toward patient	Blood refluxes into catheter	Fluid restricted by diaphragm
Clamping sequence	Clamp before disconnection	No specified clamping	Clamp after disconnection	No specified clamping
Commercial Examples	BD Smartsite	ICU Medical Microclave Clear	Braun Ultrasite	ICU Medical Neutron
	BD Q-Syte	Baxter One-Link	BD MaxPlus	Nexus TKO-5
	Baxter Interlink	RyMed Invision	Braun Caresite	Nexus TKO-6P
	ICU Medical Clave*	Nexus NIS-6P		

**Table 2:** Catheter related blood stream infection (CRBSI) associated pathogens [42].

Pathogen	Prevalence
Coagulase-negative staphylococci (i.e., *S. epidermidis*)	20.9%
*Staphylococcus aureus*	18.1%
*Escherichia coli*	7.4%
*Klebsiella pneumoniae/Klebsiella oxytoca*	9.4%
*Enterococcus faecalis*	9.1%

**Table 3:** Commercial catheter incorporating antimicrobial/antifouling features.

Manufacturer	Product	Antimicrobial Agent	Mode of Action
Kimal	Altius^®^ ProActiv+	Polyhexamethylene biguanide (PHMB)	Contact
Teleflex	ARROWg+ard^®^	Chlorhexidine and silver sulfadiazine	Eluting
	Chlorag+ard^®^	Chlorhexidine only	
Cook Medical	Spectrum^®^	Minocycline & Rifampin	Eluting
B. Braun	Certofix^®^	Polyhexamethylene biguanide (PHMB)	Contact
Edward Lifesciences	Vantex CVC Oligon	Silver ions	
	AMC Thromboshield	Benzalkonium chloride with heparin coating	Contact
